# Bridging practice and evidence: insights from the Scandinavian Society of Anaesthesiology and Intensive Care Medicine and Saudi Critical Care Society Guidelines on trauma-related VTE

**DOI:** 10.1186/s13049-026-01566-9

**Published:** 2026-01-26

**Authors:** Marwa Amer, Waleed Alhazzani, Morten Hylander Møller, Faisal A. Al-Suwaidan, Mohammed Alshahrani

**Affiliations:** 1 Medical/Critical Pharmacy Division, King Faisal Specialist Hospital and Research Center, Riyadh, Saudi Arabia; 2 College of Medicine and Pharmacy, Alfaisal University, Riyadh, Saudi Arabia; 3 Health Research Center, Ministry of Defense Health Services, Riyadh, Saudi Arabia; 4 Critical Care and Internal Medicine Department, College of Medicine, Imam Abdulrahman Bin Faisal University, Dammam, Saudi Arabia; 5 Department of Intensive Care, Copenhagen University Hospital—Rigshospitalet, Copenhagen, Denmark; 6 Department of Clinical Medicine, University of Copenhagen, Copenhagen, Denmark; 7 Division of Neurology, Department of Medicine, Security Forces Hospital, Riyadh, Saudi Arabia; 8 Neurology Clinical Lead, Ministry of Health, Riyadh, Saudi Arabia; 9 College of Medicine, Princess Nourah Bint Abdulrahman University, Riyadh, Saudi Arabia; 10 College of Medicine, Dar Al-Uloom University, Riyadh, Saudi Arabia; 11 Department of Emergency and Critical Care, King Fahd Hospital of the University, Imam Abdulrahman Bin Faisal University, Dammam, Saudi Arabia; 12 Alfaisal University- College of Medicine, King Faisal Specialist Hospital & Research Center, Al Mathar Ash Shamali, Riyadh, 11564 Saudi Arabia

**Keywords:** Trauma, Venous thromboembolism prophylaxis, ICU care, Critical care guidelines

## Abstract

**Background:**

Trauma-related venous thromboembolism (VTE) represents significant challenges in clinical care for patients with critical illnesses, highlighting the need for evidence-based recommendations. The Saudi Critical Care Society (SCCS), in collaboration with international experts, developed the “VTE Prophylaxis in Trauma Intensive Care Unit Patients” clinical practice guidelines. The guidelines were developed using the Grading of Recommendations, Assessment, Development, and Evaluation methodology and provide consensus-based, actionable recommendations tailored to diverse clinical contexts. This article highlights key aspects of the guidelines, emphasizing practical implementation strategies for trauma VTE prophylaxis management.

**Main body:**

The trauma VTE prophylaxis guidelines, endorsed by the Scandinavian Society of Anaesthesiology and Intensive Care Medicine (SSAI), focus on the timing of initiation, agent selection, and mechanical prophylaxis strategies for patients with trauma. The guidelines address unique challenges and knowledge gaps, providing adaptable strategies for clinicians in high-resource and resource-constrained settings.

**Conclusion:**

Here, we highlight key aspects of the guidelines, the importance of evidence-based practices, adherence strategies, the need for adaptability in special populations and low-resource settings, and future research priorities in trauma and critical care.

## Background

Trauma-related venous thromboembolism (VTE) in patients with critical illnesses is a key challenge in intensive care, as it contributes to significant morbidity and mortality globally [1]. These challenges highlight the urgent need for standardized, evidence-based guidelines to optimize quality of care in this high-risk population [2].

The Saudi Critical Care Society (SCCS) developed the Trauma VTE Prophylaxis Guidelines to provide a structured framework for optimizing VTE prevention in patients with trauma, addressing both standard-risk and high-risk populations [2–4]. The guidelines focus on key clinical domains, including timing of prophylaxis initiation, pharmacologic agent selection, and the use of mechanical prophylaxis strategies. These guidelines were subsequently reviewed and formally endorsed by the Scandinavian Society of Anaesthesiology and Intensive Care Medicine (SSAI). The guidelines are grounded in the Grading of Recommendations, Assessment, Development, and Evaluation (GRADE) methodology, ensuring that their recommendations are informed by high-quality evidence and structured evaluation tools, including the Population, Intervention, Comparison, and Outcome (PICO) framework [5, 6]. This systematic approach enhances transparency, standardizes decision-making, and improves the applicability of various recommendations across diverse clinical contexts [7].

Th﻿e guidelines were developed by multidisciplinary expert panels from clinical pharmacy, medicine, and critical care disciplines to emphasize the importance of collaboration and patient-centered care. By integrating robust evidence frameworks, including GRADE and PICO, we ensure the credibility, feasibility, and broad applicability of these recommendations to bridge the gaps between evidence and practice.

Here, we present key highlights of the guidelines, summarize actionable recommendations, and discuss the importance of evidence-based practices. We also propose strategies to improve adherence through standardized protocols, training programs, and multidisciplinary collaboration and address their implementation in diverse settings to emphasize adaptability to resource-constrained environments and particular populations. Finally, we call for action regarding future research to refine practices, address knowledge gaps, and optimize outcomes in trauma and critical care.

## Main text

### Key highlights of the guidelines

Table 1 summarizes key recommendations from the Trauma VTE Guidelines.

**Table 1:** Key recommendations from the Trauma Venous Thromboembolism (VTE) guidelines

Clinical scenario	Recommendation	Strength of evidence	Practical considerations
Trauma VTE guideline: the full evidence profiles and decision frameworks can be accessed here			
VTE Prophylaxis in Blunt Solid Organ Injuries	Initiate pharmacological VTE prophylaxis within 24–48 h in patients with low-risk blunt solid organ injuries who are being managed non-operatively	Conditional, very low	Exclude patients with hemodynamic instability, active bleeding, or high-grade injuries. Regularly assess bleeding risk
VTE Prophylaxis in Isolated Blunt TBI	Early pharmacological VTE prophylaxis (24–72 h post-injury) for low-risk patients with stable brain imaging and neurological examination results	Conditional, very low	Avoid patients who are considered high-risk and require neurosurgical intervention. Use mechanical prophylaxis as a bridge until stabilization
Spine Trauma or SCI	For non-operative spine trauma or SCI, initiate pharmacological VTE prophylaxis within 24–48 h. Initiate within 48 h post-fixation for surgical cases	Conditional, very low	Begin mechanical prophylaxis early for all patients with SCIs. Consider IVCFs if pharmacological options are delayed or contraindicated
Preferred Pharmacologic Agent	Prefer LMWH (e.g., enoxaparin and dalteparin) over UFH for patients with trauma who are receiving pharmacologic prophylaxis	Conditional, low	Use UFH for patients with renal impairment (e.g., creatinine clearance < 30 mL/min). Monitor for HIT
Dose of Pharmacologic VTE Prophylaxis	Use either intermediate-high dose (e.g., enoxaparin 40 mg subcutaneously every 12 h) or conventional dosing of LMWH in patients who are at low risk of bleeding	Conditional, very low	Avoid intermediate-high dosing in patients who are at high risk of bleeding (e.g., age > 65 years, weight < 50 kg, those with low creatinine clearance, or patients with TBI or SCI who are at high risk for bleeding)
Use of Mechanical Prophylaxis	Initiate mechanical prophylaxis (e.g., pneumatic compression devices) for all patients with trauma who are unable to receive pharmacologic prophylaxis	Strong, very low	Ensure proper device placement and monitor for skin breakdown or discomfort during prolonged use
Adjunct Mechanical Prophylaxis	Consider adjunct use of mechanical prophylaxis for additional protection in patients with trauma receiving pharmacologic prophylaxis	Conditional, very low	Adjunctive strategies can be tailored to patient-specific risk profiles
Routine Duplex Ultrasound Surveillance	In high-risk patients with trauma who are unable to receive pharmacologic prophylaxis, use routine bilateral lower extremity ultrasound screening to detect asymptomatic DVT	Conditional, very low	Not applicable to ambulatory patients, low-risk patients with trauma, or those with symptoms indicating diagnostic imaging
Prophylactic IVCFs	Avoid routine placement of prophylactic IVCFs in patients with trauma	Conditional, very low	Temporary retrievable IVCFs may be considered for patients who are unable to receive pharmacological VTE prophylaxis for > 7 days (e.g., because of ongoing bleeding risk)

*Abbreviations*: *DVT* deep venous thrombosis, *HIT* heparin-induced thrombocytopenia, *IBW* ideal body weight, *IVCFs* inferior vena cava filters, *LMWH* low-molecular-weight heparin, *SCI* spinal cord injury, *TBI* traumatic brain injury, *UFH* unfractionated heparin

#### Risk stratification and prophylaxis timing

Personalized risk stratification is the cornerstone of VTE prevention in patients with trauma.

- Clinical Practice Point: Utilize validated tools, including the Caprini score or Padua Prediction Score, to assess VTE risk and tailor prophylaxis accordingly.
- Timing Recommendation: Initiate pharmacologic prophylaxis with low-molecular-weight heparin (LMWH) within 24–48 h of trauma stabilization, provided bleeding risks are controlled. For high-risk cases, use mechanical prophylaxis (intermittent pneumatic compression [IPC] devices) until pharmacological options are feasible [8].

#### Pharmacological prophylaxis

LMWH is considered the first-line agent because of its favorable safety profile and efficacy in preventing VTE (conditional recommendation, low certainty of evidence) [9].

Dosing Adjustments: Adjust doses for weight and renal function; monitor anti-Xa levels in patients with obesity or critical illnesses [10].

#### Mechanical prophylaxis

IPC devices serve as effective alternatives for patients with contraindications for pharmacologic prophylaxis (strong recommendation, very low certainty of evidence) [1, 2].

Practical Tip: Ensure proper device application and consider sequential compression devices for enhanced efficacy.

#### Special populations

- Solid Organ Injuries: Early initiation (24–48 h post-injury) is recommended in adults with blunt solid organ injuries to the liver, spleen, or kidney who are managed nonoperatively and have controlled bleeding or low hemorrhagic risk, as this strategy has been associated with reduced VTE events without increased failure of nonoperative management or bleeding complications [11–13]. Conversely, in patients at high risk of major bleeding—including those with high-grade solid organ injuries, large hemoperitoneum, or ongoing transfusion requirements—pharmacologic prophylaxis should be deferred until hemostasis and physiological stability are confirmed, with mechanical prophylaxis used as a bridging strategy during this period (conditional recommendation, very low certainty of evidence) [14, 15].
- Traumatic Brain Injury (TBI): Prophylaxis initiation requires confirmation of radiographic stability (24–72 h post-injury) [16]. Recent large-scale data from the Trauma Quality Improvement Program database demonstrate significant inter-hospital variation in the timing of VTE pharmacologic prophylaxis among patients with TBI, with earlier initiation (24–48 h) correlating with lower adjusted trauma and TBI-related mortality [17]. Despite the historical hesitation to initiate pharmacologic prophylaxis early in TBI due to bleeding risk, early VTE pharmacologic prophylaxis was not associated with higher hemorrhagic complications in this cohort of patients with non-neurosurgical TBI. Observational and propensity-matched cohort studies have demonstrated that initiation within 24–72 h of injury is associated with lower rates of VTE without increased risk of intracranial hemorrhage progression, repeat neurosurgical intervention, or mortality [18, 19]. Therefore, the SCCS recommendation to consider VTE pharmacologic prophylaxis within 24–72 h (conditional recommendation, very low certainty of evidence) based on repeat imaging and clinical stability aligns with high-performing trauma center practices and can be emphasized as a quality measure in regional trauma systems.
- Spinal Cord Injuries (SCI): Early initiation (24–48 h post-injury) is advised in adults with isolated spine trauma or fracture and/or SCI with low bleeding risk, who are managed non-operatively, thereby balancing hematoma risk with VTE prevention (conditional recommendation, very low certainty of evidence) [20, 21].

#### Inferior vena cava filters

The guideline discourages routine use of inferior vena cava filters for primary prophylaxis due to potential complications and limited evidence of survival benefits. Selective use is recommended for patients with VTE who have contraindications to anticoagulation, accompanied by structured follow-up to ensure retrieval and minimize complications (conditional recommendation, very low certainty of evidence) [22].

#### Ultrasound surveillance

Routine duplex ultrasonography surveillance in patients with trauma remains controversial, and practice varies substantially across trauma systems. While some centers continue scheduled (e.g., weekly) screening of selected high-risk, asymptomatic patients—particularly when pharmacologic prophylaxis is delayed or contraindicated—routine surveillance is not recommended for most patients with trauma. Consistent with our guidelines’ recommendation, a selective approach may be considered for patients at elevated VTE risk who are not candidates for pharmacologic prophylaxis (conditional recommendation; very low certainty of evidence) [1, 2]. This recommendation reflects the limited and methodologically heterogeneous evidence base, which consists largely of single-center observational studies and one randomized trial showing reduced in-hospital pulmonary embolism (PE) without a demonstrable mortality benefit [23].

Routine surveillance substantially increases detection of asymptomatic or distal deep vein thrombosis, raising concern for overdiagnosis and potential downstream anticoagulation-related harm without clear improvement in patient-important outcomes. In this context, the potential benefits of surveillance should be weighed against surveillance bias, increased detection of clinically uncertain events, and uncertain impact on patient-important outcomes, particularly PE [24–26]. Diagnostic ultrasonography should remain reserved for patients with clinical signs or symptoms of deep vein thrombosis, and routine surveillance should be avoided in ambulatory or low-risk patients.

### Bridging global and local practices

The guidelines integrate global evidence with region-specific considerations, ensuring practical applicability across diverse healthcare environments. While aligned with international standards, including those of the Western Trauma Association (WTA) and the American Association for the Surgery of Trauma (AAST) Critical Care Committee Clinical Consensus Document, the SCCS guidelines prioritize flexibility to address unique challenges in high- and low-resource settings [25, 26]. Table 2 compares the SCCS guidelines to those of the WTA and AAST. The WTA algorithms and AAST Critical Care Committee Clinical Consensus Document emphasize expert-driven consensus and pragmatic decision-making, whereas the SCCS guideline prioritizes formal evidence certainty grading and explicit benefit–harm trade-offs through GRADE.

**Table 2:** Comparison of trauma-related VTE prophylaxis recommendations across SCCS, WTA, and AAST guidance

Aspect	SCCS Guidelines (Endorsed by SSAI)	WTA algorithms	AAST critical care committee clinical consensus document
Agent Selection	Recommend LMWH over UFH as the preferred pharmacological agent (conditional recommendation, low certainty of evidence)	Recommend LMWH over UFH based on strong evidence for VTE prevention and reduced risk of HIT	Recommends LMWH over UFH based on similar evidence
Standard LMWH Dose	Advocate for either intermediate–high dose LMWH (enoxaparin 40 mg every 12 h) or conventional dosing LMWH (conditional recommendation, very low certainty of evidence)	Recommend 40 mg of LMWH every 12 h to achieve target anti-factor Xa levels, which is higher than older recommendations (30 mg every 12 h)	Recommends 40 mg of LMWH every 12 h for most patients
Timing of Initiation	Emphasize early initiation, ideally within 24–48 h of trauma stabilization if bleeding risks permit (conditional recommendation, very low certainty of evidence)	Recommend initiation within 24 h of injury if bleeding is controlled	Recommends immediate initiation for patients without active bleeding or high-risk injuries; otherwise, delays depend on specific conditions
High-Risk Injuries	Highlight special considerations for TBI, SCI, and solid organ injuries with tailored timing	Recommend starting within 24 h of bleeding cessation or stabilization of injuries	Recommends using Modified Berne–Norwood Criteria for TBI and starting within 48 h for spinal cord or solid organ injuries
Prophylaxis Dose in High-Risk Populations	Suggest lower initial doses for patients with high-risk injuries, particularly TBI, SCI, and high risk of bleeding (conditional recommendation, very low certainty of evidence)	Recommend a lower dose (30 mg LMWH every 12 h) for high-risk injuries, reflecting safety concerns with higher doses	Follows similar dosing for high-risk populations (30 mg every 12 h)
Missed Doses	Stress the importance of continuous thromboprophylaxis, minimizing interruptions unless clinically unavoidable	Emphasize continuous prophylaxis without interruption. Missed doses significantly increase VTE risk	Stresses continuous prophylaxis and advises against holding LMWH for most surgical procedures, except spinal fixation
Dose Adjustments	Advocate for adjusting LMWH doses based on weight, anti-Xa levels, renal function, and specific populations	Discuss weight-based dosing (0.5 mg/kg every 12 h) and adjustments to achieve target anti-Xa levels	Recommends similar weight-based dosing and anti-Xa adjustments. Additionally, emphasizes adjustments for renal impairment
Special Populations	Suggest dose adjustments for older patients, those with low body weight, pregnant individuals, and those with renal impairment	Recommend lower dosing (30 mg every 12 h) for CrCl 30–60 mL/min, older patients (aged > 65 years), those with low body weight (< 50 kg), and pregnant patients	Similar recommendations for these populations, adding the use of UFH for those with CrCl values of < 30 mL/min

*Abbreviations*: *AAST* American Association for the Surgery of Trauma, *CrCl* Creatinine Clearance, *HIT* heparin-induced thrombocytopenia, *LMWH* low-molecular-weight heparin, *SCI* spinal cord injury, *SCCS* Saudi Critical Care Society, *TBI* traumatic brain injury, *UFH* unfractionated heparin, *VTE* venous thromboembolism, *WTA* Western Trauma Association

## Discussion

### Role of evidence-based guidelines in trauma management

Evidence-based guidelines are central to optimizing trauma care in the setting of profound patient heterogeneity, competing risks of thrombosis and bleeding, and time-critical decision-making. The SCCS guidelines for VTE prophylaxis in patients with trauma provide structured, actionable recommendations grounded in systematically appraised evidence and transparent benefit–harm assessment [1, 2]. By explicitly prioritizing patient safety, feasibility, and resource stewardship, the guidelines offer a pragmatic framework for navigating complex clinical scenarios frequently encountered in trauma intensive care units (ICUs). These priorities closely align with Scandinavian trauma and critical care systems, which emphasize equity, data-driven practice, and continuous quality improvement supported by national registries and standardized care pathways.

### Methodological considerations in trauma guidelines development

The conditional nature of many trauma-related VTE prophylaxis recommendations reflects fundamental methodological limitations within the trauma evidence base rather than a lack of clinical importance or equipoise. A recent systematic review of randomized trials in traumatic hemorrhage demonstrated frequent overestimation of treatment effects, inaccurate baseline risk assumptions, and high rates of futility-driven trial termination, undermining confidence in effect estimates and limiting the certainty of downstream recommendations [27]. These challenges help explain why many widely adopted trauma practices continue to rest on low- or very low-certainty evidence.

This methodological fragility is particularly evident in the literature addressing the timing of pharmacologic VTE prophylaxis in TBI. Despite decades of observational research, the optimal initiation strategy remains unresolved—not because of contradictory findings, but due to pervasive bias, heterogeneity, and lack of clinically meaningful endpoints. A contemporary systematic review identified 21 studies evaluating prophylaxis timing in adult TBI; 20 were judged to be at critical risk of bias using the ROBINS-I tool, and none provided high-certainty evidence applicable to patients with severe TBI or those undergoing neurosurgical intervention [28]. Substantial heterogeneity in definitions of “early” prophylaxis, injury severity classification, radiographic stability criteria, pharmacologic agents, and neurosurgical management precluded meta-analysis and limited generalizability. Consequently, the field remains unable to definitively answer the core clinical question: when is it safe to initiate pharmacologic prophylaxis in TBI?

These evidence limitations carry important clinical consequences. Concern regarding intracranial hemorrhage progression remains a dominant driver of delayed prophylaxis, particularly in patients with severe injuries, evolving radiographic findings, or recent neurosurgical procedures. Although observational data increasingly suggest that earlier prophylaxis may be feasible in carefully selected low-risk patients, these data lack the granularity required to inform decision-making in higher-risk scenarios.

From a guideline methodology perspective, this uncertainty explains why contemporary trauma guidelines, including the SCCS recommendations endorsed by the SSAI, deliberately avoid rigid, time-based initiation thresholds for VTE prophylaxis in TBI. Instead, recommendations emphasize radiographic stability on repeat computed tomography and neurological stability on clinical examination as the most reliable anchors for clinical judgment. This stability-based approach acknowledges the low certainty of evidence, accommodates biological heterogeneity, and aligns with Parkland- and Berne–Norwood-style risk stratification frameworks, prioritizing patient safety over arbitrary temporal targets.

### Implementation and adherence to guideline challenges

Despite the availability of comprehensive, methodologically robust guidelines, real-world adherence to trauma-related VTE prophylaxis recommendations remains inconsistent, driven by challenges in dissemination, education, and bedside implementation [29]. Persistent practice variation reflects not only operational barriers but also the unresolved nature of the underlying evidence, particularly in high-risk populations such as patients with TBI.

A large contemporary survey of trauma surgeons illustrates this evidence–practice gap. Although more than 90% of respondents reported an intention to initiate pharmacologic prophylaxis within 72 h of TBI, self-reported adherence to institutional protocols ranged from only 25% to 75% of eligible patients [30]. Common drivers of deviation included concern for intracranial hemorrhage progression, disagreement between trauma and neurosurgical teams, and clinical scenarios insufficiently addressed by existing guidelines. These findings highlight a critical tension between guideline intent and bedside decision-making, wherein clinicians are compelled to rely on individualized judgment in the absence of high-certainty evidence—particularly for patients with severe TBI or following neurosurgical intervention [31]. In this context, guideline recommendations emphasizing radiographic stability, neurological examination, and multidisciplinary consensus—rather than rigid time-based thresholds—represent a pragmatic and methodologically defensible response to uncertainty. Such recommendations acknowledge residual evidence gaps while providing clinically meaningful anchors for decision-making in complex and evolving trauma scenarios.

Importantly, non-adherence is not limited to decisions regarding when to start prophylaxis. Even after pharmacologic VTE prophylaxis is prescribed, missed doses of LMWH represent a frequent and clinically meaningful implementation failure. Evidence from implementation studies indicates that most missed doses are attributable to patient refusal rather than clinical contraindications, highlighting that adherence challenges extend beyond clinician decision-making to patient understanding and engagement [32]. These failures contribute to preventable variation in care delivery despite nominal guideline compliance.

### Enhancing adherence and making guidelines implementable

Improving adherence to trauma VTE prophylaxis guidelines requires translation of recommendations into reliable clinical workflows rather than further expansion of the evidence base [33–35].

**Fig. 1:**
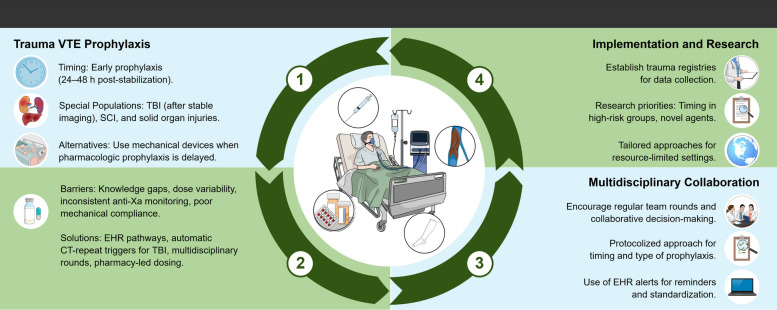
Summary of key insights and implementation priorities. The figure provides a visual synthesis of how the guidelines translate into real-world ICU care and highlights their adaptability across diverse clinical contexts. • Trauma VTE Prophylaxis: Emphasizes early pharmacologic prophylaxis (within 24–48 h post-stabilization), with special considerations for high-risk populations such as those with TBI, SCI, and solid organ injuries. Mechanical prophylaxis is recommended when pharmacologic options are delayed or contraindicated. • Multidisciplinary Collaboration: Encourages structured team communication, protocol-driven prophylaxis, and integration of electronic health record (EHR) alerts to standardize practice. • Implementation and Research: Calls for establishing trauma registries, prioritizing research in high-risk groups, and tailoring guidelines for resource-limited settings. Abbreviations: VTE – Venous thromboembolism; TBI – Traumatic brain injury; SCI – Spinal cord injury; EHR – Electronic health record; CT – Computed tomography; anti-Xa – Anti–factor Xa (activity/assay)

Figure 1 illustrates algorithmic decision-making pathways used in trauma-related VTE prophylaxis guidelines, emphasizing injury severity, radiographic stability, and neurological assessment to enhance bedside applicability.

Several implementation-focused strategies have demonstrated effectiveness in reducing preventable practice variation:

1. Targeted Education and Communication: Patient-centered education interventions, combined with nurse-focused training, directly address a common cause of missed prophylaxis doses—patient refusal. Implementation studies demonstrate that structured education bundles supported by real-time electronic prompts reduced missed LMWH doses from 12.9% to 9.3% and decreased patient refusal by approximately 50%, without altering pharmacologic strategy. These findings emphasize that communication quality, rather than drug selection or dosing, is a primary determinant of successful prophylaxis delivery.
2. Standardized, Electronic Health Record (EHR)-Integrated Protocols: Institution-wide protocols aligned with guideline recommendations simplify decision-making and reduce unwarranted variation. Embedding these protocols into EHRs through order sets, reminders, and automated reassessment prompts supports consistent execution once prophylaxis is deemed safe to initiate. Beyond protocol embedding, system-level strategies to address missed or delayed prophylaxis are gaining attention. A pragmatic randomized clinical trial protocol describes an artificial intelligence (AI)–driven, EHR-integrated clinical decision support “nudge” that identifies hospitalized patients at high risk for hospital-acquired VTE who are not receiving prophylaxis and prompts reconsideration of prophylaxis orders [36]. Although this approach is not trauma-specific and outcome data are pending, it illustrates how AI-enabled decision support may complement standardized EHR protocols by improving adherence and reducing gaps in prophylaxis delivery; trauma-specific validation and effectiveness data remain necessary before such systems can inform guideline recommendations.
3. Contextual and Regional Adaptation: Guideline implementability depends on alignment with local infrastructure and resources. In resource-limited settings, mechanical prophylaxis may serve as an interim strategy when pharmacologic options are delayed or unavailable. In Scandinavian trauma systems, high digital maturity, multidisciplinary ICU teams, and national quality registries—such as the Swedish Intensive Care Registry and the Danish Trauma Database—enable real-time monitoring of adherence, feedback, and continuous quality improvement. Within Saudi trauma systems, pragmatic approaches include embedding nurse-facing prompts and brief education scripts into EHRs; tracking missed-dose rates as a core VTE quality metric; and delivering concise patient-facing messages explaining the rationale for LMWH, the persistence of post-trauma VTE risk, and the safety of prophylaxis once imaging stability is confirmed.
4. Interdisciplinary Collaboration: Close collaboration among pharmacists, intensivists, anesthesiologists, surgeons, and nurses mitigates implementation challenges by enabling shared risk assessment and coordinated decision-making. Routine multidisciplinary rounds allow dynamic reassessment of bleeding and thrombotic risk and support individualized, high-fidelity application of guideline recommendations—a practice increasingly adopted within Scandinavian and Saudi ICU cultures [37, 38].

## Future research directions

Although the guidelines are comprehensive, critical knowledge gaps remain, particularly in high-risk subgroups and post-acute phases of care, where current recommendations rely largely on low- or very low-certainty evidence (Tables 3 and 4). Future research should prioritize studies that enroll under-represented trauma phenotypes, use standardized and clinically meaningful definitions and endpoints, and incorporate realistic baseline-risk estimates and effect-size assumptions to reduce trial futility and improve interpretability.

1. High-risk injury patterns and timing decisions
  TBI: Prospective studies are needed in patients with severe TBI, especially those undergoing neurosurgical interventions (e.g., craniotomy, external ventricular drain placement, intracranial pressure monitoring), who remain systematically under-represented in existing datasets. Future trials should standardize definitions of radiographic stability, neurological stability, and clinically meaningful hemorrhage progression to enable reproducible, patient-centered risk stratification. Comparative effectiveness studies should evaluate stability-based initiation strategies versus fixed time-based thresholds using outcomes that reflect both safety and benefit (intracranial hemorrhage progression, VTE events, neurosurgical reintervention, and mortality) [39].
  SCI: Studies should determine whether earlier prophylaxis (< 48 h) confers clinically meaningful benefit across injury severity strata, including prospective risk stratification by neurological impairment (e.g., ASIA grade) and surgical versus non-surgical pathways.
  Solid Organ Injury: Evidence remains limited for penetrating trauma and high-grade injuries; future studies should explicitly examine these subgroups and report standardized bleeding and re-intervention outcomes to support safer generalization of timing recommendations.
  Cross-Cutting Priority: Across TBI and SCI populations, the field would benefit from harmonized definitions of “early” prophylaxis, standardized imaging-stability criteria, and consistent reporting of baseline risk to support pooled analyses and guideline certainty upgrades.
2. Dose optimization and monitoring
  Dosing Strategies: Comparative trials should evaluate weight-based dosing, anti-Xa–guided adjustment, and viscoelastic testing–informed approaches using patient-important outcomes (symptomatic VTE, bleeding requiring intervention, mortality), rather than surrogate endpoints alone.
3. Novel agents and evolving anticoagulant strategies
  Novel Agents: Non-heparin strategies remain investigational in trauma. Aspirin may warrant evaluation in carefully selected, lower-risk post-acute pathways where LMWH is not feasible; however, robust trauma-specific evidence is limited [40]. Direct oral anticoagulants (DOACs) raise important safety and reversibility concerns in bleeding-prone phenotypes (e.g., TBI) and should be avoided until trauma-specific safety data are available.
  Factor XI/XIa Inhibition: Emerging agents targeting factor XI or XIa (e.g., abelacimab, osocimab, milvexian) have shown reduced bleeding compared with DOACs in non-trauma populations; however, they have not been evaluated in acute trauma, TBI, or solid organ injury [41]. Accordingly, LMWH remains the foundation of pharmacologic prophylaxis in trauma care, and factor XI inhibitors should be regarded as investigational until trauma-specific trials establish safety and net clinical benefit.
4. Post-discharge prophylaxis and late events
  An under-recognized gap in trauma-related VTE prevention is the persistence of thrombotic risk after hospital discharge, with a substantial proportion of events occurring within the first three months following injury. Randomized trauma-specific trials are needed to define which phenotypes benefit from extended prophylaxis, optimal duration, and the safest regimens. Until such evidence is available, future studies should focus on defining risk thresholds (e.g., pelvic/lower-extremity fractures, SCI, prolonged immobility), bleeding-risk stratification, and patient-centered outcomes (symptomatic VTE, readmissions, patient-reported burden, and adherence) [42, 43].
5. Economic evaluation and trial efficiency
  Cost-effectiveness analyses should be embedded within future trauma VTE research to evaluate economic viability across high-resource and resource-limited settings, including the incremental value of dose-adjustment strategies and post-discharge prophylaxis pathways. Scandinavian centers with experience in adaptive platform trial infrastructure (e.g., INCEPT) are well-positioned to support efficient multicenter evaluation using pragmatic enrollment and registry-enabled follow-up where feasible [44]. Future trials should also incorporate prognostic enrichment, realistic baseline-risk estimates, and clinically meaningful endpoints to strengthen the evidentiary foundation underpinning guideline updates.

**Table 3:** Novel therapeutic medications for Venous Thromboembolism Prophylaxis (VTE)

Therapeutic agent	Mechanism of action	Potential benefits	Clinical considerations
DOACs	Inhibit specific clotting factors (e.g., Factor Xa and thrombin)	- Fixed dosing and no routine monitoring - Oral administration	- Limited data in populations with trauma - Contraindicated in active bleeding or unstable injuries - Availability and cost of reversal agents . Drug interactions with anticonvulsants in TBI
Aspirin	Inhibits platelet cyclooxygenase, reducing thromboxane A2 production and platelet aggregation	- Simple administration - Potentially lower bleeding risk - Cost-effective for low-risk patients (Some orthopedic data available after a fracture) particularly in resource-constrained environments	- Limited efficacy in high-risk patients with trauma - Not a substitute for anticoagulation in severe cases
Factor XI / XIa Inhibitors	Selective inhibition of factor XI or activated factor XIa, attenuating thrombin generation through the intrinsic pathway while preserving hemostasis mediated by the extrinsic pathway	- Promising bleeding-sparing signals in selected non-trauma populations	- No clinical data in trauma populations, including patients with TBI, solid organ injury, or active bleeding risk - Safety, timing, reversibility, and net clinical benefits in trauma remain unknown -LMWH remains the foundation of pharmacologic VTE prophylaxis in trauma

*Abbreviations*: *DOACs* direct oral anticoagulants, *LMWH* low-molecular-weight heparin, *TBI* traumatic brain injury

**Table 4:** Summary of strategies, findings, recommendations, and gaps in trauma-related VTE Prophylaxis

Aspect	Keyfindings	Recommendations	Research gaps
Current Practices	- LMWH is the preferred agent for pharmacological VTE prophylaxis - Fixed dosing regimens (e.g., 30 or 40 mg two times daily) - Mechanical prophylaxis using intermittent pneumatic compression devices for patients at high bleeding risk - Delayed initiation in high-risk patients (e.g., those with TBIs or solid organ injuries)	- Continue the use of LMWH as a first-line agent - Implement fixed dosing with weight-based adjustments for patients with high body mass indexes - Utilize mechanical prophylaxis where pharmacological prophylaxis is contraindicated	- Data comparing the effectiveness and safety of DOACs versus LMWH in trauma patients remain sparse, particularly among bleeding-prone phenotypes such as those with TBIs - Limited data on the long-term impact of extended prophylaxis in patients with trauma
Solid Organ Injuries	Early VTE prophylaxis (< 48 h) post-injury reduces VTE risk without increasing bleeding or failure of non-operative management	- Initiate prophylaxis within 48 h in patients with blunt solid organ injuries being managed non-operatively, after confirming hemodynamic and radiographic stability	- Limited evidence for high-grade injuries and penetrating trauma regarding safe prophylaxis timing
TBI	LMWH post-radiographic stability (24–48 h) reduces VTE risk and may improve mortality	- Confirm stability of intracranial bleeding before initiating prophylaxis within 48 h, using repeat imaging and clinical assessment	- Limited data on heterogeneous TBI presentations, neurosurgical subgroups, and optimal timing for varied clinical scenarios
SCI	Early initiation (< 72 h) is beneficial; evidence supports earlier use (< 48 h) in stable cases	- Tailor prophylaxis based on injury level (cervical versus thoracic versus lumbar) and completeness of injury (ASIA classification)	- Limited prospective data to define optimal timing thresholds across SCI severity, surgical pathways, and bleeding-risk strata
Implementation Strategies	- Education campaigns targeting clinicians to improve awareness - Real-time electronic alerts and reminders for timely prophylaxis - Audit and feedback loops to monitor adherence - Multidisciplinary rounds to tailor personalized patient care	- Standardize education campaigns for hospital-wide implementation - Integrate electronic reminders into existing EHR systems - Foster collaboration through regular multidisciplinary meetings	- Limited studies on long-term retention of knowledge from education programs - Inadequate data on the scalability of audit processes in low-resource settings - Few studies on the cost-effectiveness of these interventions
Patient-Centered Interventions	Personalized education and involvement in care decisions improve acceptance of prophylaxis	- Develop patient education materials and involve patients in prophylaxis planning, particularly for those who refuse injections such as LMWH	- Sparse data on the effectiveness of patient-centered approaches in trauma-specific VTE prevention
Tailored Interventions for High-Risk Populations	Injury-specific, tailored protocols may reduce practice variability and improve safety in patients with TBIs, SCIs, and solid organ injuries	- Create tailored prophylaxis plans that incorporate injury patterns, timing and agent selection based on individual risks	- Limited robust data for standardizing dose adjustments and initiation timing in high-risk populations
Cost-Effectiveness Studies	Evaluating the financial implications of implementation strategies can justify investments in adherence-improving interventions	- Conduct cost-effectiveness studies focusing on low-resource settings - Include economic evaluations in VTE research protocols to provide evidence for scaling successful interventions	- Minimal evidence from low-resource settings on economic feasibility - Lack of comprehensive cost–benefit analyses comparing strategies such as electronic alerts and extended prophylaxis

*Abbreviations*: *DOACs* direct oral anticoagulants, *EHR* electronic health record, *LMWH* low-molecular-weight heparin, *SCI* spinal cord injury, *TBI* traumatic brain injury, *UFH* unfractionated heparin, *VTE* venous thromboembolism

### Evolving models of guideline development

Traditional clinical practice guidelines remain the cornerstone of evidence-based medicine because of their comprehensive scope, multidisciplinary consensus processes, and rigorous application of the GRADE framework. By addressing multiple PICO questions through full systematic reviews, these guidelines enable transparent balancing of benefits, harms, values, and resource considerations. However, their development is inherently resource-intensive, time-consuming, and dependent on sustained volunteer engagement, rendering them vulnerable to obsolescence in rapidly evolving clinical domains such as trauma and critical care [45].

To address these limitations, alternative guideline models have emerged. Rapid practice guidelines prioritize timeliness by focusing on narrowly defined, practice-changing clinical questions, enabling faster translation into care at the expense of breadth. Living guidelines extend this approach through continuous evidence surveillance and iterative updating but require substantial methodological infrastructure, stable funding, and coordinated expertise to remain sustainable. Each model, therefore, reflects a trade-off between scope, agility, and resource requirements.

Despite these differences, the GRADE framework remains foundational to trustworthy guideline development and clinical credibility. Hybrid approaches that integrate traditional GRADE methodology with streamlined evidence synthesis, prioritized question selection, and early engagement of end users may be particularly well-suited to trauma care, where evidence evolves incrementally, and implementation feasibility is as critical as statistical certainty [46].

### Toward personalized VTE prophylaxis in trauma care

Beyond classical immobility-related risk factors, trauma induces a delayed prothrombotic phenotype characterized by platelet hyperreactivity, fibrinolysis shutdown, endothelial dysfunction, and immunothrombotic pathways, providing biological plausibility for sustained venous thromboembolism risk despite resolution of early bleeding concerns [47].

Against this biologically heterogeneous backdrop**,** most contemporary clinical practice guidelines are based on average treatment effects derived from randomized trials; however, these averages may not reflect individual patient responses—a phenomenon known as heterogeneity of treatment effects [48]. This limitation is particularly salient in trauma and critical care, where patients vary widely in injury patterns, bleeding risk, inflammatory response, and dynamic coagulation profiles.

Emerging work in critical care demonstrates that phenotype-informed strategies—such as individualized oxygen targets based on predicted treatment effects—may outperform population-level recommendations [49]. Translating similar principles to trauma-related VTE prophylaxis represents an important conceptual advance toward personalized thromboprophylaxis, particularly in populations where both thrombotic and hemorrhagic risks evolve rapidly over time.

Viscoelastic hemostatic testing can identify trauma-associated hypercoagulable phenotypes such as fibrinolysis shutdown, which are consistently associated with increased VTE risk [47]. While these modalities are well-established for guiding transfusion and hemorrhage management, current evidence is insufficient to support their routine use for individualizing pharmacologic VTE prophylaxis, and such applications should be regarded as investigational, given the predominance of observational data, heterogeneous definitions of hypercoagulability, and limited predictive performance for patient-important thrombotic outcomes [50].

Beyond viscoelastic hemostatic testing, multi-omics and biomarker-based approaches offer additional promise for individualized risk stratification. Early metabolomic and proteomic studies have demonstrated strong discriminatory performance for thrombotic risk prediction, although external validation and demonstration of clinical utility are still required before clinical adoption [51]. In trauma-specific cohorts, proteomic signatures distinguishing patients who develop VTE despite standard prophylaxis are beginning to reveal pathways related to coagulation, innate immunity, and endothelial activation, supporting future development of biomarker-informed strategies while remaining investigational at present [52].

Advances in digital health and AI may further support personalized and timely VTE prevention. AI-assisted radiology workflows have already reduced time to detect incidental PE, and future real-time risk prediction models integrating imaging, laboratory data, and clinical variables may eventually support adaptive, patient-centered decisions regarding prophylaxis initiation and escalation [53]. Collectively, these developments signal a shift from static, population-based recommendations toward dynamic, individualized thromboprophylaxis paradigms.

### Call to action: “strengthening trauma research and implementation infrastructure”

Addressing persistent evidence and implementation gaps in trauma care will require sustained investment in research infrastructure and coordinated international collaboration [54]. Partnerships among funding agencies, professional societies, and healthcare institutions are essential to enable multicenter trials, facilitate data sharing, and accelerate innovation.

The SCCS–SSAI collaboration provides a strong foundation for advancing trauma care globally, emphasizing equity, contextual adaptability, and patient-centered outcomes. Nordic countries benefit from mature national trauma registries and critical care databases that support benchmarking, policy refinement, and data-driven practice improvement. Saudi Arabia and other Middle East and North Africa (MENA) countries are actively developing similar infrastructures, presenting a strategic opportunity to align registry development with guideline implementation and research priorities.

Establishing national ICU trauma registries with standardized data elements will be pivotal for tracking injury patterns, interventions, outcomes, and guideline adherence in real time. Such platforms—modelled in part on Scandinavian registry systems—can support continuous quality improvement and enable future multinational trauma trials linking MENA and Nordic institutions [55].

### Contextualizing recommendations: ADOLOPMENT in MENA and Low- and Middle-Income Countries (LMIC) settings

The guidelines were intentionally designed for adaptability across diverse healthcare environments and were jointly developed by experts from Saudi Arabia and Nordic countries—two regions with distinct infrastructure and care-delivery models. This collaboration enhances external validity and real-world applicability.

In high-resource settings, advanced monitoring tools and comprehensive training facilitate full implementation, including anti-Xa–guided LMWH dosing. Conversely, resource-constrained environments may require simplified protocols, such as prioritizing mechanical prophylaxis when pharmacologic options or monitoring are unavailable. These context-sensitive adaptations support equitable delivery of evidence-based care.

The ADOLOPMENT framework provides a pragmatic pathway to operationalize such adaptations. By enabling health systems to adopt, adapt, or develop de novo recommendations based on local capacity and needs, ADOLOPMENT preserves methodological integrity while promoting feasibility and cultural relevance. This approach empowers national societies, policymakers, and frontline clinicians to align international standards with local realities, fostering sustainable critical care practices.

### Addressing implementation barriers in the MENA context

Implementation of clinical practice guidelines across the MENA region is frequently hindered by systemic and environmental barriers, including limited resources, inadequate training and dissemination strategies, and concerns regarding guideline trustworthiness. Regional evidence and Delphi consensus studies further highlight clinician reliance on experiential practice, limited leadership support, and insufficient contextualization as dominant obstacles [56].

Importantly, experience from LMIC trauma registry initiatives indicates that implementation failure most commonly occurs at the level of information management, including data capture, completeness, and governance [57]. Effective mitigation strategies include standardized data-collection tools, early stakeholder engagement, protected data-management roles, and sustainable multi-site funding models linked to quality-improvement deliverables.

Top-ranked facilitators consistently include awareness and education, point-of-care access to guidelines, local customization, and strong organizational endorsement. These findings reinforce the importance of early stakeholder engagement, institutional alignment, and integration of guideline training into broader health-system improvement agendas. Collaborations such as the SCCS–SSAI initiative exemplify how Scandinavian trauma systems can support scalable implementation while promoting contextual validation across diverse care environments.

## Conclusions

The guidelines represent a significant advancement in trauma management, providing evidence-based recommendations to address key clinical practice challenges. By emphasizing risk stratification, early intervention, and personalized care, the guidelines aim to optimize patient outcomes and promote standardized practices across diverse healthcare settings. A key strength of the guidelines is their joint development by the SCCS and SSAI, bringing together diverse healthcare perspectives and enhancing their generalizability to various populations. This collaboration ensures that the recommendations are grounded in globally relevant evidence while remaining adaptable to local contexts.

The guidelines highlight the importance of multidisciplinary collaboration and adaptable protocols to address the varying needs of high- and low-resource settings. Implementation strategies that prioritize education, protocol standardization, and teamwork are essential for ensuring equitable and effective adoption.

Future iterations of the guidelines should be guided by robust research, particularly in under-represented regions, to fill knowledge gaps and refine recommendations. Establishing trauma registries and fostering sustained investment in critical care research represent crucial steps toward generating data-driven insights and improving global trauma care.

This call to action encourages the broader adoption of evidence-based practices, expansion of research, and commitment to education to ensure that patients with critical illnesses receive high-quality, patient-centered care that bridges the gap between evidence and practice.

## Data Availability

All data included in the final manuscript are available in the original published guidelines.

## Abbreviations

AAST: American Association for the Surgery of Trauma
ARDS: Acute respiratory distress syndrome
AI: Artificial intelligence
CPGs: Clinical practice guidelines
DOAC: Direct oral anticoagulant
EHRs: Electronic health records
GRADE: Grading of Recommendations, Assessment, Development, and Evaluation
ICU: Intensive care unit
IPC: Intermittent pneumatic compression
LMICs: Low- and middle-income countries
LMWH: Low-molecular-weight heparin
MENA: Middle East and North Africa
PE: Pulmonary embolism
PICO: Population, Intervention, Comparison, and Outcome
RPG: Rapid Practice Guidelines
SCCS: Saudi Critical Care Society
SCI: Spinal Cord Injuries
SSAI: Scandinavian Society of Anaesthesiology and Intensive Care Medicine
TBI: Traumatic Brain Injury
VTE: Venous thromboembolism
WTA: Western Trauma Association

## References

[1] Dhillon NK, Barmparas G, Lin TL, Linaval NT, Yang AR, Sekhon HK, et al. A systems-based approach to reduce deep venous thrombosis and pulmonary embolism in trauma patients. World J Surg. 2021;45(3):738–45. 10.1007/s00268-020-05849-9. 33169176 10.1007/s00268-020-05849-9

[2] Amer M, Alshahrani MS, Arabi YM, Al-Jedai A, Alshaqaq HM, Al-Sharydah A, et al. Saudi Critical Care Society clinical practice guidelines on the prevention of venous thromboembolism in adults with trauma: reviewed for evidence-based integrity and endorsed by the Scandinavian Society of Anaesthesiology and Intensive Care Medicine. Ann Intensive Care. 2023;13(1):41. 10.1186/s13613-023-01135-8. 37165105 10.1186/s13613-023-01135-8 PMC10172441

[3] Amer M, Bawazeer M, Maghrabi K, Amin R, De Vol E, Hijazi M. Timing and dose of pharmacological thromboprophylaxis in adult trauma patients: perceptions, barriers, and experience of Saudi Arabia practicing physicians. Saudi Surg J. 2020;8(2):67–81. 10.4103/ssj.ssj_50_20.

[4] Sigurðsson MI, Chew MS, Olkkola KT, Rehn M, Kalliomäki ML, Møller MH. Saudi Critical Care Society clinical practice guidelines on the prevention of venous thromboembolism in adults with trauma: endorsement by the Scandinavian Society of Anaesthesiology and Intensive Care Medicine. Acta Anaesthesiol Scand. 2023;67(9):1288–90. 10.1111/aas.14292. 37280639 10.1111/aas.14292

[5] Møller MH, Alhazzani W, Oczkowski S, Belley-Cote E, Haney M. Intensive care medicine rapid practice guidelines in Acta Anaesthesiologica Scandinavica. Acta Anaesthesiol Scand. 2023;67(5):566–8. 10.1111/aas.14215. 36794852 10.1111/aas.14215

[6] Alhazzani W, Møller MH, Belley-Cote E, Citerio G. Intensive care medicine rapid practice guidelines (ICM-RPG): paving the road of the future. Intensive Care Med. 2019;45(11):1639–41. 10.1007/s00134-019-05786-9. 31552444 10.1007/s00134-019-05786-9

[7] Alhazzani W, Lewis K, Jaeschke R, Rochwerg B, Møller MH, Evans L, et al. Conflicts of interest disclosure forms and management in critical care clinical practice guidelines. Intensive Care Med. 2018;44(10):1691–8. 10.1007/s00134-018-5367-6. 30264380 10.1007/s00134-018-5367-6

[8] Hecht JP, Han EJ, Cain-Nielsen AH, Scott JW, Hemmila MR, Wahl WL. Association of timing of initiation of pharmacologic venous thromboembolism prophylaxis with outcomes in trauma patients. J Trauma Acute Care Surg. 2021;90(1):54–63. 10.1097/TA.0000000000002912. 32890341 10.1097/TA.0000000000002912

[9] Tran A, Fernando SM, Carrier M, Siegal DM, Inaba K, Vogt K, et al. Efficacy and safety of low molecular weight heparin versus unfractionated heparin for prevention of venous thromboembolism in trauma patients: a systematic review and meta-analysis. Ann Surg. 2022;275(1):19–28. 10.1097/SLA.0000000000005157. 34387202 10.1097/SLA.0000000000005157

[10] Taylor A, Huang E, Waller J, White C, Martinez-Quinones P, Robinson T. Achievement of goal anti-Xa activity with weight-based enoxaparin dosing for venous thromboembolism prophylaxis in trauma patients. Pharmacotherapy. 2021;41(6):508–14. 10.1002/phar.2526. 33864688 10.1002/phar.2526

[11] Schellenberg M, Owattanapanich N, Emigh B, Van Gent JM, Egodage T, Murphy PB, et al. When is it safe to start venous thromboembolism prophylaxis after blunt solid organ injury? A prospective American Association for the Surgery of Trauma multi-institutional trial. J Trauma Acute Care Surg. 2024;96(2):209–15. 10.1097/TA.0000000000004163. 37872669 10.1097/TA.0000000000004163

[12] Ashley JR, Burczak KW, Cotton BA, Clements TW. Management of blunt abdominal trauma. Br J Surg. 2024;111(7):znae168. 10.1093/bjs/znae168. 39030780 10.1093/bjs/znae168 PMC11492274

[13] Cioffi SP, Cimbanassi S, Chiara O. Blunt abdominal trauma: watch and wait. Curr Opin Crit Care. 2023;29(6):674–81. 10.1097/MCC.0000000000001095. 37861213 10.1097/MCC.0000000000001095

[14] Skarupa D, Hanna K, Zeeshan M, Madbak F, Hamidi M, Haddadin Z, et al. Is early chemical thromboprophylaxis in patients with solid organ injury a solid decision? J Trauma Acute Care Surg. 2019;87(5):1104–12. 10.1097/TA.0000000000002438. 31299694 10.1097/TA.0000000000002438

[15] Murphy PB, de Moya M, Karam B, Menard L, Holder E, Inaba K, et al. Optimal timing of venous thromboembolic chemoprophylaxis initiation following blunt solid organ injury: meta-analysis and systematic review. Eur J Trauma Emerg Surg. 2022;48(3):2039–46. 10.1007/s00068-021-01783-0. 34537859 10.1007/s00068-021-01783-0

[16] Störmann P, Osinloye W, Freiman TM, Seifert V, Marzi I, Lustenberger T. Early chemical thromboprophylaxis does not increase the risk of intracranial hematoma progression in patients with isolated severe traumatic brain injury. World J Surg. 2019;43(11):2804–11. 10.1007/s00268-019-05072-1. 31267142 10.1007/s00268-019-05072-1

[17] Coaston TN, Vadlakonda A, Shen A, Balian J, Cho NY, Benharash P, et al. Thromboembolism prophylaxis timing is associated with center mortality in traumatic brain injury: a Trauma Quality Improvement Program retrospective analysis. J Trauma Acute Care Surg. 2025;98(3):468–75. 10.1097/TA.0000000000004469. 39560961 10.1097/TA.0000000000004469

[18] Byrne JP, Mason SA, Gomez D, Hoeft C, Subacius H, Xiong W, et al. Timing of pharmacologic venous thromboembolism prophylaxis in severe traumatic brain injury: a propensity-matched cohort study. J Am Coll Surg. 2016;223(4):621-631.e5. 10.1016/j.jamcollsurg.2016.06.382. 27453296 10.1016/j.jamcollsurg.2016.06.382

[19] Byrne JP, Witiw CD, Schuster JM, Pascual JL, Cannon JW, Martin ND, et al. Association of venous thromboembolism prophylaxis after neurosurgical intervention for traumatic brain injury with thromboembolic complications, repeated neurosurgery, and mortality. JAMA Surg. 2022;157(3):e215794. 10.1001/jamasurg.2021.5794. 34910096 10.1001/jamasurg.2021.5794 PMC8674806

[20] Banerjee O, Rodrigues R, Adkins L, Busl KM. Venous thromboembolism prophylaxis in the neurocritically ill population. J Clin Med. 2025;14(13):4434. 10.3390/jcm14134434. 40648808 10.3390/jcm14134434 PMC12249765

[21] Godat LN, Haut ER, Moore EE, Knudson MM, Costantini TW. Venous thromboembolism risk after spinal cord injury: a secondary analysis of the CLOTT study. J Trauma Acute Care Surg. 2023;94(1):23–9. 10.1097/TA.0000000000003807. 36203245 10.1097/TA.0000000000003807

[22] Alshaqaq HM, Al-Sharydah AM, Alshahrani MS, Alqahtani SM, Amer M. Prophylactic inferior vena cava filters for venous thromboembolism in adults with trauma: an updated systematic review and meta-analysis. J Intensive Care Med. 2023;38(6):491–510. 10.1177/08850666231163141. 36939472 10.1177/08850666231163141

[23] Kay AB, Morris DS, Woller SC, Stevens SM, Bledsoe JR, Lloyd JF, et al. Trauma patients at risk for venous thromboembolism who undergo routine duplex ultrasound screening experience fewer pulmonary emboli: a prospective randomized trial. J Trauma Acute Care Surg. 2021;90(5):787–96. 10.1097/TA.0000000000003104. 33560104 10.1097/TA.0000000000003104

[24] Al-Sharydah AM, Alshahrani MS, Maghrabi K, Tashkandi W, Amer M. Ultrasound surveillance for deep venous thrombosis and subsequent venous thromboembolism in adults with trauma: a systematic review and meta-analysis. Medicine. 2023;102(43):e35625. 10.1097/MD.0000000000035625. 37904393 10.1097/MD.0000000000035625 PMC10615543

[25] Rappold JF, Sheppard FR, Carmichael SP, Cuschieri J, Ley E, Rangel E, et al. Venous thromboembolism prophylaxis in the trauma intensive care unit: an American Association for the Surgery of Trauma Critical Care Committee Clinical Consensus Document. Trauma Surg Acute Care Open. 2021;6(1):e000643. 10.1136/tsaco-2020-000643. 10.1136/tsaco-2020-000643 PMC7908288 33718615

[26] Ley EJ, Brown CV, Moore EE, Sava JA, Peck K, Ciesla DJ, et al. Updated guidelines to reduce venous thromboembolism in trauma patients: a Western Trauma Association critical decisions algorithm. J Trauma Acute Care Surg. 2020;89(5):971–81. 10.1097/TA.0000000000002830. 32590563 10.1097/TA.0000000000002830 PMC7587238

[27] Ghossein J, Fernando SM, Rochwerg B, Inaba K, Lampron J, Tran A. A systematic review and meta-analysis of sample size methodology for traumatic hemorrhage trials. J Trauma Acute Care Surg. 2023;94(6):870–6. 10.1097/TA.0000000000003944. 36879398 10.1097/TA.0000000000003944

[28] Stubbs J, Byrne JP. Mind the implementation gap: departure from national guidelines for thromboprophylaxis in traumatic brain injury highlights an urgent need for better evidence. Trauma Surg Acute Care Open. 2025;10(4):e002118. 10.1136/tsaco-2025-002118. 41262855 10.1136/tsaco-2025-002118 PMC12625917

[29] Ratnasekera A, Geerts W, Haut ER, Price M, Costantini T, Murphy P. Implementation science approaches to optimizing venous thromboembolism prevention in patients with traumatic injuries: findings from the 2022 consensus conference to implement optimal venous thromboembolism prophylaxis in trauma. J Trauma Acute Care Surg. 2023;94(3):490–4. 10.1097/TA.0000000000003850. 36729882 10.1097/TA.0000000000003850 PMC9974883

[30] Alexander KM, Butts CC, Lee YL, Kutcher ME, Polite N, Haut ER, et al. Survey of venous thromboembolism prophylaxis in trauma patients: current prescribing practices and concordance with clinical practice guidelines. Trauma Surg Acute Care Open. 2023;8(1):e001070. 10.1136/tsaco-2022-001070. 37205274 10.1136/tsaco-2022-001070 PMC10186479

[31] Fiorentino MN, Ratnasekera A. Venous thromboembolism prophylaxis initiation in adults with traumatic brain injury: elusive and hard to pin down. Trauma Surg Acute Care Open. 2025;10(2):e001942. 10.1136/tsaco-2025-001942. 40557260 10.1136/tsaco-2025-001942 PMC12186030

[32] Haut ER, Owodunni OP, Shaffer DL, McQuigg D, Samuel D, Hobson DB, et al. Implementing a patient-centered education bundle to improve venous thromboembolism prevention. JAMA Surg. 2025;160(12):1326–32. 10.1001/jamasurg.2025.4136. 41060638 10.1001/jamasurg.2025.4136 PMC12509082

[33] Schellenberg M, Costantini T, Joseph B, Price MA, Bernard AC, Haut ER. Timing of venous thromboembolism prophylaxis initiation after injury: findings from the consensus conference to implement optimal VTE prophylaxis in trauma. J Trauma Acute Care Surg. 2023;94(3):484–9. 10.1097/TA.0000000000003847. 36729602 10.1097/TA.0000000000003847 PMC9970012

[34] Teichman AL, Cotton BA, Byrne J, Dhillon NK, Berndtson AE, Price MA, et al. Approaches for optimizing venous thromboembolism prevention in injured patients: findings from the consensus conference to implement optimal venous thromboembolism prophylaxis in trauma. J Trauma Acute Care Surg. 2023;94(3):469–78. 10.1097/TA.0000000000003854. 36729884 10.1097/TA.0000000000003854 PMC9975027

[35] Sarkies MN, Jones LK, Gidding SS, Watts GF. Improving clinical practice guidelines with implementation science. Nat Rev Cardiol. 2022;19(1):3–4. 10.1038/s41569-021-00645-x. 34799708 10.1038/s41569-021-00645-x

[36] Walsh CG, Long Y, Novak LL, Salwei ME, Tillman B, French B, et al. AI-driven clinical decision support to reduce hospital-acquired venous thromboembolism: a trial protocol. JAMA Netw Open. 2025;8(10):e2535137. 10.1001/jamanetworkopen.2025.35137. 41042513 10.1001/jamanetworkopen.2025.35137 PMC12495493

[37] Haut ER, Byrne JP, Price MA, Bixby P, Bulger EM, Lake L, et al. Proceedings from the 2022 consensus conference to implement optimal venous thromboembolism prophylaxis in trauma. J Trauma Acute Care Surg. 2023;94(3):461–8. 10.1097/TA.0000000000003843. 36534056 10.1097/TA.0000000000003843 PMC9974764

[38] Dhillon NK, Haut ER, Price MA, Costantini TW, Teichman AL, Cotton BA, et al. Novel therapeutic medications for venous thromboembolism prevention in trauma patients: findings from the Consensus Conference to Implement Optimal venous thromboembolism prophylaxis in Trauma. J Trauma Acute Care Surg. 2023;94(3):479–83. 10.1097/TA.0000000000003853. 36729880 10.1097/TA.0000000000003853 PMC9974825

[39] Tignanelli CJ, Shah S, Vock D, Siegel L, Serrano C, Haut E, et al. A pragmatic, stepped-wedge, hybrid type II trial of interoperable clinical decision support to improve venous thromboembolism prophylaxis for patients with traumatic brain injury. Implement Sci. 2024;19(1):57. 10.1186/s13012-024-01386-4. 39103955 10.1186/s13012-024-01386-4 PMC11299263

[40] Major Extremity Trauma Research Consortium (METRC); O’Toole RV, Stein DM, O’Hara NN, Frey KP, Taylor TJ et al. Aspirin or low-molecular-weight heparin for thromboprophylaxis after a fracture. N Engl J Med. 2023;388(3):203–13. 10.1056/NEJMoa2205973. 10.1056/NEJMoa2205973 36652352

[41] Bedrouni W, Bedrouni M, Douketis JD. New horizons in venous thromboembolism management: a narrative review. J Clin Med. 2025;14(21):7668. 10.3390/jcm14217668. 41227064 10.3390/jcm14217668 PMC12609676

[42] Knudson MM, Ikossi DG, Khaw L, Morabito D, Speetzen LS. Thromboembolism after trauma: an analysis of 1602 episodes from the American College of Surgeons National Trauma Data Bank. Ann Surg. 2004;240(3):490–6. 10.1097/01.sla.0000137138.40116.6c. 15319720 10.1097/01.sla.0000137138.40116.6c PMC1356439

[43] Berndtson AE, Cross A, Yorkgitis BK, Kennedy R, Kochuba MP, Tignanelli C, et al. American Association for the Surgery of Trauma/American College of Surgeons Committee on Trauma Clinical protocol for postdischarge venous thromboembolism prophylaxis after trauma. J Trauma Acute Care Surg. 2024;96(6):980–5. 10.1097/TA.0000000000004307. 38523134 10.1097/TA.0000000000004307

[44] Granholm A, Møller MH, Kaas-Hansen BS, Jensen AK, Munch MW, Kjær MN, et al. INCEPT: the Intensive Care Platform Trial-Design and protocol. Acta Anaesthesiol Scand. 2025;69(4):e70023. 10.1111/aas.70023. 10.1111/aas.70023 PMC11907384 40084471

[45] Wiersinga WJ, Møller MH, de Waele JJ, Prescott HC. On the future of guidelines. Crit Care. 2025;29(1):465. 10.1186/s13054-025-05731-x. 41176597 10.1186/s13054-025-05731-x PMC12579812

[46] Schinkel M, van der Poll T, Wiersinga WJ. Artificial intelligence for early sepsis detection: a word of caution. Am J Respir Crit Care Med. 2023;207(7):853–4. 10.1164/rccm.202212-2284VP. 36724366 10.1164/rccm.202212-2284VP PMC10111986

[47] Reed CR, Curry N, Juffermans NP, Neal MD. Hemostatic abnormalities after trauma resuscitation: challenges and strategies in caring for the critically injured patient. Ann Intensive Care. 2025;15(1):163. 10.1186/s13613-025-01587-0. 41107643 10.1186/s13613-025-01587-0 PMC12534623

[48] Munroe ES, Spicer A, Castellvi-Font A, Zalucky A, Dianti J, Graham Linck E, et al. Evidence-based personalised medicine in critical care: a framework for quantifying and applying individualised treatment effects in patients who are critically ill. Lancet Respir Med. 2025;13(6):556–68. 10.1016/S2213-2600(25)00054-2. 40250459 10.1016/S2213-2600(25)00054-2 PMC12362495

[49] Buell KG, Spicer AB, Casey JD, Seitz KP, Qian ET, Graham Linck EJ, et al. Individualized treatment effects of oxygen targets in mechanically ventilated critically ill adults. JAMA. 2024;331(14):1195–204. 10.1001/jama.2024.2933. 38501205 10.1001/jama.2024.2933 PMC10951851

[50] Rossetto A, Wohlgemut JM, Davenport R. Viscoelastic testing in trauma: not all that glitters is a gold standard. Trauma Surg Acute Care Open. 2025;10(2):e001919. 10.1136/tsaco-2025-001919. 40510862 10.1136/tsaco-2025-001919 PMC12161410

[51] Febra C, Saraiva J, Vaz F, Macedo J, Al-Hroub HM, Semreen MH, et al. Acute venous thromboembolism plasma and red blood cell metabolomic profiling reveals potential new early diagnostic biomarkers: observational clinical study. J Transl Med. 2024;22(1):200. 10.1186/s12967-024-04883-8. 38402378 10.1186/s12967-024-04883-8 PMC10894498

[52] LaCroix IS, Dzieciatkowska M, Cendali F, Sanders K, Wade CE, Cotton BA, et al. Multiomics reveal plasma constituents associated with thrombosis among trauma patients who do not respond to enoxaparin. Blood Vessels Thrombosis Hemostasis. 2025;2(4):100102. 10.1016/j.bvth.2025.100102. 10.1016/j.bvth.2025.100102 PMC12594916 41210697

[53] Soffer S, Klang E, Shimon O, et al. Deep learning for pulmonary embolism detection on computed tomography pulmonary angiogram: a systematic review and meta-analysis. Sci Rep. 2021;11:15814. 10.1038/s41598-021-95249-3. 34349191 10.1038/s41598-021-95249-3 PMC8338977

[54] Arabi YM, Al-Dorzi HM, AlQahtani S, Al Aseri Z, Aldekhyl S, Al Duhailib Z, et al. Critical care research in Saudi Arabia: onward and upward. Saudi Crit Care J. 2023;7(3):49–52. 10.4103/sccj.sccj_18_23.

[55] Amer M, Møller MH, Granholm A, Alotaibi HF, AlMuhaidib S, Al Duhailib Z, et al. A systematic methodological evaluation of sepsis guidelines: protocol for quality assessment and consistency of recommendations. Acta Anaesthesiol Scand. 2025;69(6):e70036. 10.1111/aas.70036. 40357564 10.1111/aas.70036 PMC12070244

[56] Almazrou SH, Alfaifi SI, Alfaifi SH, Hakami LE, Al-Aqeel SA. Barriers to and facilitators of adherence to clinical practice guidelines in the Middle East and North Africa region: a systematic review. Healthcare. 2020;8(4):564. 10.3390/healthcare8040564. 33333843 10.3390/healthcare8040564 PMC7765264

[57] Cote MP, Alty IG, Hamzah R, Tripathi I, Montalvan A, Salim A, et al. Barriers and facilitators of trauma registries in low- and middle-income countries: a scoping review. J Trauma Acute Care Surg. 2025;99(6):975–89. 10.1097/TA.0000000000004759. 41325093 10.1097/TA.0000000000004759

